# An atypical arrhythmia

**DOI:** 10.1007/s12471-013-0403-1

**Published:** 2013-04-09

**Authors:** A. A. M. Wilde

**Affiliations:** Cardioloog AMC Amsterdam, Department of Cardiology, Academic Medical Centre, Meibergdreef 9, 1105 AZ Amsterdam, the Netherlands

**Fig. 1:**
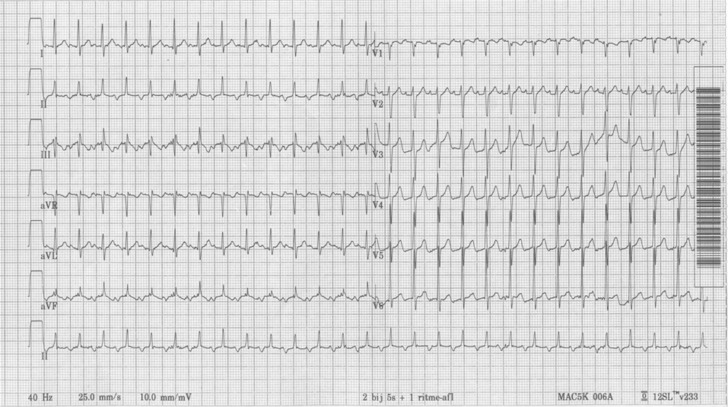
ECG at presentation

**Fig. 2:**
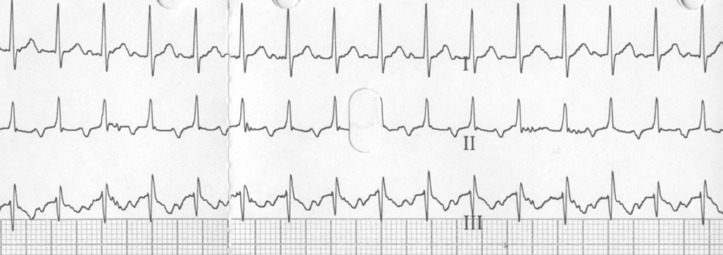
An enlargement of leads I, II and III at a different point in time

A 58-year-old male presented with palpitations, which he had experienced occasionally in the past years, but this time it did not stop. His ECG at presentation is shown in Fig. 1. Physical examination revealed no abnormalities. Figure 2 shows an enlargement of leads I, II and III at a different point in time. What is your diagnosis?

**Answer**

You will find the answer elsewhere in this issue.

